# New Synthetic Isoxazole Derivatives Acting as Potent Inducers of Fetal Hemoglobin in Erythroid Precursor Cells Isolated from β-Thalassemic Patients

**DOI:** 10.3390/molecules29010008

**Published:** 2023-12-19

**Authors:** Cristina Zuccato, Lucia Carmela Cosenza, Chiara Tupini, Alessia Finotti, Gianni Sacchetti, Daniele Simoni, Roberto Gambari, Ilaria Lampronti

**Affiliations:** 1 Department of Life Sciences and Biotechnology, Section of Biochemistry and Molecular Biology, Ferrara University, 44121 Ferrara, Italy; cristina.zuccato@unife.it (C.Z.); luciacarmela.cosenza@unife.it (L.C.C.); chiara.tupini@unife.it (C.T.); alessia.finotti@unife.it (A.F.); gianni.sacchetti@unife.it (G.S.); 2 Center “Chiara Gemmo and Elio Zago” for the Research on Thalassemia, University of Ferrara, 44121 Ferrara, Italy; 3 Department of Chemical, Pharmaceutical and Agricultural Sciences, Ferrara University, 44121 Ferrara, Italy; daniele.simoni@unife.it

**Keywords:** β-thalassemia, sickle cell disease, SCD, isoxazole derivatives, heat shock protein (HSP) inhibitors, fetal hemoglobin, HbF, erythroid differentiation, HbF inducers, γ-globin mRNA induction

## Abstract

Induction of fetal hemoglobin (HbF) is highly beneficial for patients carrying β-thalassemia, and novel HbF inducers are highly needed. Here, we describe a new class of promising HbF inducers characterized by an isoxazole chemical skeleton and obtained through modification of two natural molecules, geldanamycin and radicicol. After preliminary biological assays based on benzidine staining and RT-qPCR conducted on human erythroleukemic K562 cells, we employed erythroid precursors cells (ErPCs) isolated from β-thalassemic patients. ErPCs weretreated with appropriate concentrations of isoxazole derivatives. The accumulation of globin mRNAs was studied by RT-qPCR, and hemoglobin production by HPLC. We demonstrated the high efficacy of isozaxoles in inducing HbF. Most of these derivatives displayed an activity similar to that observed using known HbF inducers, such as hydroxyurea (HU) or rapamycin; some of the analyzed compounds were able to induce HbF with more efficiency than HU. All the compounds were active in reducing the excess of free α-globin in treated ErPCs. All the compounds displayed a lack of genotoxicity. These novel isoxazoles deserve further pre-clinical study aimed at verifying whether they are suitable for the development of therapeutic protocols for β-thalassemia.

## 1. Introduction

The β-thalassemias are genetically heterogenous, hereditary hematological diseases characterized by low or absent production of adult hemoglobin (HbA) [1,2,3,4]. Currently, the first therapeutic option for β-thalassemia patients is based on blood transfusion and iron-chelation therapy [4]. At present, clinical trials are ongoing using gene therapy [5] and gene-editing [6] approaches.

One therapeutic strategy to reduce the need for red blood cell transfusions is the induction of γ-globin chain through chemical inducers, with the aim to stimulate the production of fetal hemoglobin (HbF) [7,8,9,10]. It is expected that this approach would also improve the α-globin chain imbalance and thus decrease the severity of anemia in β-thalassemia patients [11,12]. In recent years, much effort has been made to identify new drug treatments which can promote the expression of fetal γ-globin genes and increase the synthesis of HbF [8]. This HbF induction strategy is not only of interest for the management of β-thalassemia patients, but also for patients affected by other hematological diseases, such as sickle-cells disease (SCD) [13,14].

Some chemotherapeutic agents, for example 5-azacytidine [15], mithramycin (MTH) [16], butyrates [17], hydroxyurea (HU) [18], and rapamycin [19,20], have been studied for their ability to enhance HbF production when administered to erythroid cells singularly or in combination. However, most of these currently identified HbF-inducing agents exhibit low efficacy and specificity, myelotoxicity, and carcinogenesis, as well as modest responses to treatment, which greatly limit their usefulness in the clinical practice [9].

At present, the only drug approved for γ-globin induction is HU, of which efficacy and safety are still unresolved issues [21,22]. Until now, this drug has been used in thalassemia intermedia and SCD. It acts through multiple mechanisms and performs its cytotoxic activity by accelerating the differentiation process and stimulating cellular stress response pathways, leading to an overall increase in the number of F cells [21,22]. More recently, decitabine [23] and HQK-1001 [24] have been proposed as new fetal globin inducers under pre-clinical and clinical development.

In a very informative paper, Flanagan et al. studied, by microarray expression analysis and RT-qPCR validation, HU activity on early reticulocytes isolated from children with SCA enrolled in the NCT00305175 clinical trial [25]. In this study, CD71+ reticulocytes were examined in in order to identify novel genes affected by HU in erythroid cells in vivo. An interesting finding was that HU treatment was associated with a down-regulation of the heat shock protein (HSP) HSP90AA1 gene. This was among the most down-regulated genes when both microarray and RT-qPCR data were considered [25]. Accordingly, the rationale of our study was based on the hypothesis that HSP90 down-regulation (including that induced by HSP90 inhibitors) might be associated with the phenotype “HbF induction” characterized by treatment of erythroid cells with hydroxyurea [25,26].

In order to develop innovative HbF inducers, new 3,4-isoxazolediamides are studied here; these compounds were designed and synthesized by introducing modifications in the original structures of geldanamycin and radicicol [26,27,28], known natural HSP inhibitors, and therefore potential inducers of HbF in erythroid cells from β-thalassemia patients [25,26]. Apart from the interest in finding novel highly effective HbF inducers, the structures of geldanamycin and radicicol were deeply modified [29] because both of them exhibit several problems related to possible use in therapy, such as poor solubility, significant hepatotoxicity, and intrinsic chemical instability or deprivation of in vivo activity. This novel class of synthetic compounds, containing the isoxazole nucleus, showed potent and selective inhibition of Hsp90 [30].

**Figure 1:**
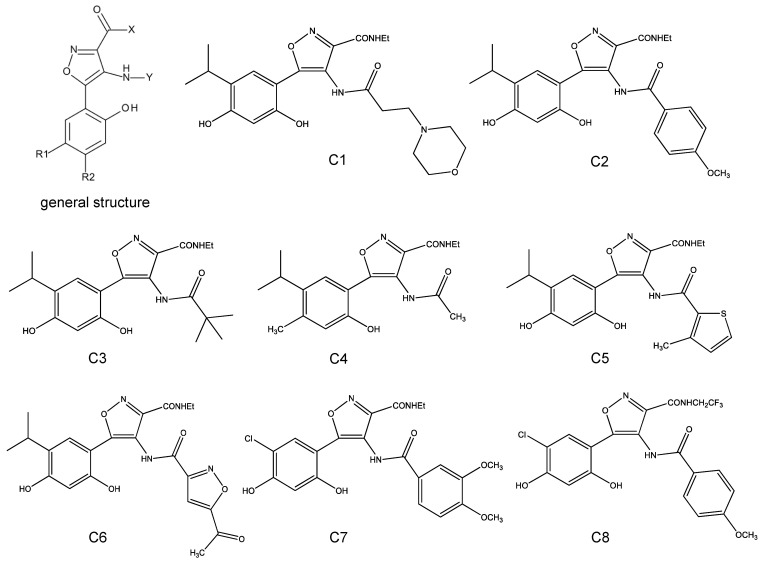
General structure (**left**) and chemical formula of the 3,4-isoxazolediamides (derivatives 1–8). R1: isopropyl (**c1**, **c2**, **c3**, **c4**, **c5**, **c6**) or chloride (**c7** and **c8**); R2: hydroxy (**c1**, **c2**, **c3**, **c5**, **c6**, **c7**, **c8**), or methyl (**c4**); X: ethyl (**c1**, **c2**, **c3**, **c4**, **c5**, **c6**, **c7**) or 2,2,2-trifluoroethyl (**c8**); Y: 4-ethylmorpholine (**c1**), para-methoxy benzoyl, (**c2**), tert-buthyl (**c3**), methyl (**c4**), 3-methyl-thienyl (**c5**), 5-acethyl-isoxazolyl (**c6**), 3,4 dimethoxy-benzoyl (**c7**) or para-methoxy benzoyl (**c8**).

We found in a preliminary study that these new isoxazole derivatives (Figure 1), characterized by a resorcinolic portion and two amide moieties, showed high HSP90 inhibition and capability in inducing erythroid differentiation [31]. Here, we extend the analysis on K562 cells [32,33] to the expression of embryo–fetal globin genes, and the effects of these promising compounds were verified on γ-globin gene expression and HbF production in erythroid precursors cells (ErPCs) derived from β-thalassemia patients with different genotypes and diverse starting levels of HbF [16,19].

## 2. Results

The chemical structures of the new isoxazole derivatives (characterized by a resorcinolic portion and two amide moieties) are shown in Figure 1. The synthesis and characterization of the isoxazole derivatives can be found in the papers by Baruchello et al. [29], Baruchello et al. [30], and Lampronti et al. [31]. The purity of these compounds was always higher than 95% [29,30,31].

### 2.1. Effects of the 3,4-Isoxazolediamine Derivatives on the Globin Gene Expression in K562 Cells

**Figure 2:**
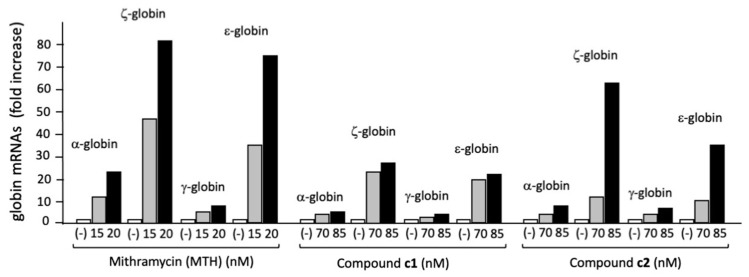
Fold increases in α, ζ, γ, and ε globin mRNA accumulation in K562 cells treated with two different concentrations of MTH were compared with the fold increase in untreated cells (-).

The data acquired by the RT-qPCR analyses allow for quantifying the increase in globin transcripts obtained following treatment of the K562 cell line through informative histograms (Figure 2).

The results of RT-qPCR concerning treatment of K562 cells with different concentrations of mithramycin (MTH, used as a positive control), allow for a comparison of the effects of the selected isoxazole derivatives compounds with those of a well-known γ-globin inducer. The range of the selected concentrations is based on the previously reported IC_50_ exhibited by mithramycin (MTH) on this cell line [31].

Figure 2 shows the fold increases in α, ζ, γ, and ε-globin mRNAs in K562 cells treated with 70 and 85 µM of derivatives **1** and **2**. MTH was used as a reference HbF inducer, as it is known to be one of the most powerful inducers of erythroid differentiation in human leukemia K562 cells [31,34,35]. The results obtained clearly indicate an induction of the embryo–fetal ζ, γ, and ε globin mRNAs, despite this being to an extent lower than that found using MTH. The treatment with the compounds **1** and **2** led to a considerable increase in globin mRNAs, both at 70 and at 85 nM concentrations.

### 2.2. Effects of 3,4-Isoxazolediamine Derivatives on Human Erythroid Precursor Cells: Recruitment of β-Thalassemia Patients and Study Design

**Figure 3:**
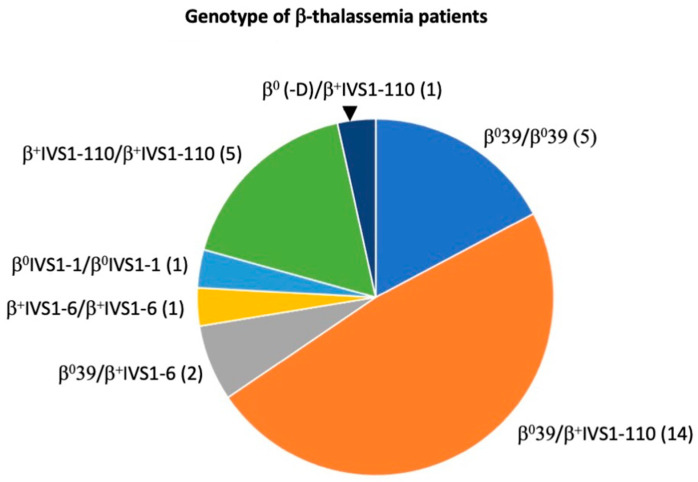
Genotype of the 29 β-thalassemia patients recruited for this study.

Early erythroid committed precursor cells (ErPCs), derived from the peripheral blood of 23 β-thalassemic patients with different genotypes (Figure 3), proliferate and differentiate into late progenitors (erythroid colony-forming units, CFU-Es) during the phase 1 culture (in the absence of erythropoietin). In phase 2 (in the presence of erythropoietin), the latter cells continue to proliferate and mature into hemoglobin-containing orthochromatic normoblasts [16,19]. The effects of the isoxazole derivatives on the cell growth and differentiation of ErPCs were determined by employing the two-phase liquid culture system and treating the cells for five days with different concentration of the selected compounds. The concentrations used for the following analysis were based on the IC_50_ value found in the K562 cell line, which was used in pilot experiments for evaluating the anti-proliferative effects of all compounds, according to a previously published procedure [31]. The isoxazole derivatives were tested on ErPCs with those concentrations and also much higher, showing their low toxicity in these cell cultures.

#### 2.2.1. Recruitment of the β-Thalassemia Patients

Figure 3 shows the genotypes of the recruited β-thalassemia patients, indicating that most of them were β^0^39/β^+^IVS-I-110. As expected [36,37], the ErPCs isolated from these patients were highly heterogeneous with respect to the basal production of HbF.

In this study we employed HU and sirolimus as reference HbF inducers for two reasons: (a) HU is currently employed by many research groups and in clinical therapy for HbF induction in β-thalassemia and sickle-cell disease (SCD) patients [8,18], and (b) rapamycin (sirolimus) has been proposed for two clinical trials in β-thalassemia, NCT03877809 (A Personalized Medicine Approach for β-thalassemia Transfusion Dependent Patients: Testing sirolimus in a First Pilot Clinical Trial) and NCT04247750 (Treatment of β-thalassemia Patients with Rapamycin (Sirolimus): From Pre-clinical Research to a Clinical Trial) [37].

#### 2.2.2. Design of the Study

**Figure 4:**
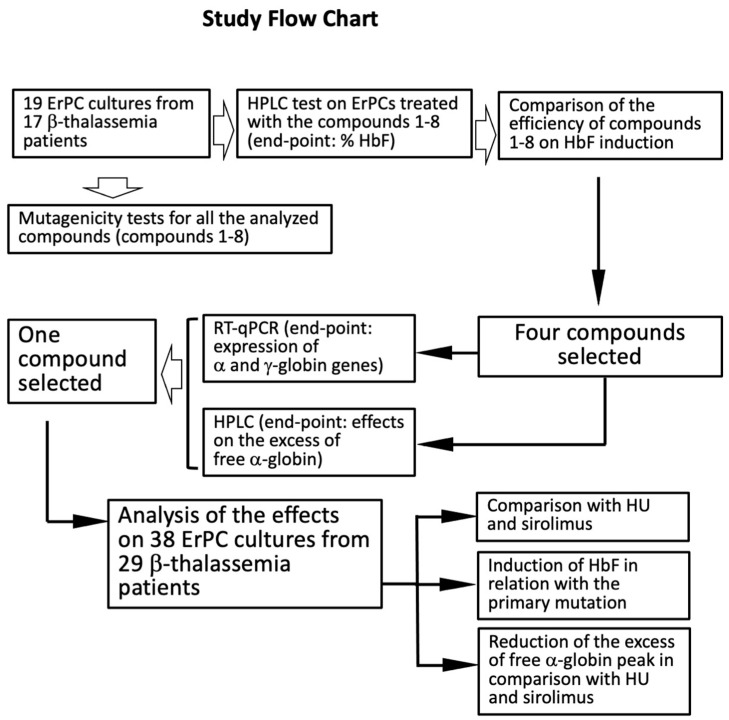
Flow chart summarizing the key steps of this study concerning the use of erythroid precursor cells from β-thalassemia patients.

A flow chart summarizing the key steps of our study is presented in Figure 4. Mutagenic tests were performed for all the compounds and a lack of mutagenic effects was demonstrated (Supplementary Tables S1–S10).

We started with induction of 19 ErPC cultures from 17 β-thalassemia patients in order to verify the effects of isoxazole derivatives on HbF production. The efficiency of compounds **c1–c8** was comparatively analyzed and the four most active compounds were selected (**c3**, **c4**, **c5,** and **c6**). In addition to an increase in HbF, expression of α-globin and β-globin mRNA was analyzed by RT-qPCR and the possible reduction in the excess of free α-globin chains analyzed by HPLC. After these analyses, one compound was selected and further characterized on 38 ErPC cultures from 29 β-thalassemia patients. For this compound the following studies were conducted: (a) comparison with HU and rapamycin (sirolimus) with respect to the efficiency of induction of HbF, considering also the primary mutations (see Figure 3); (c) reduction in the excess of free α-globin chains in comparison with HU and rapamycin.

### 2.3. Effects of 3,4-Isoxazolediamine Derivatives on Human Erythroid Precursor Cells: Isoxazole Derivatives Are Potent Inducers of γ-Globin Gene Expression and Increased Production of HbF

**Figure 5:**
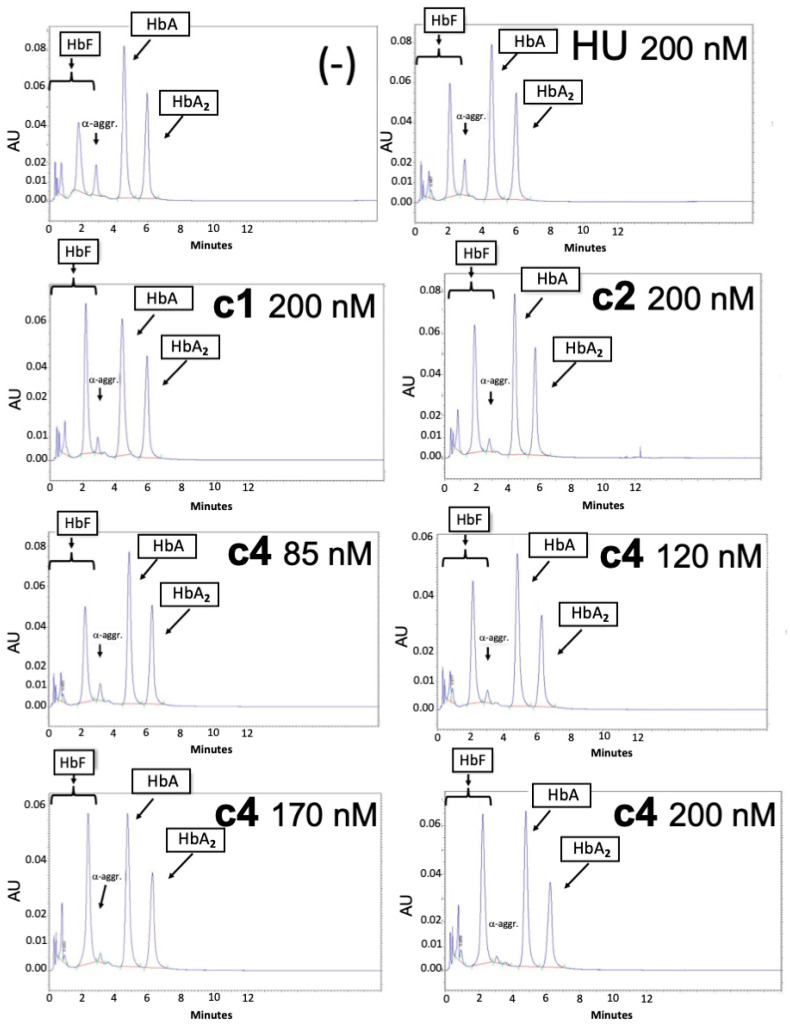
Representative HPLC patterns of cultures isolated from one β-thalassemia patient (β^0^39/β^+^ISVI-110) and HbF induction by treatments with 200 nM hydroxyurea (HU) or with the isoxazole compounds **c1** (200 nM), **c2** (200 nM), and **c4** (concentrations indicated) compared with the untreated cells (-). HbF, HbA, HbA_2_, and the α-globin peak (α aggregates) are indicated.

**Figure 6:**
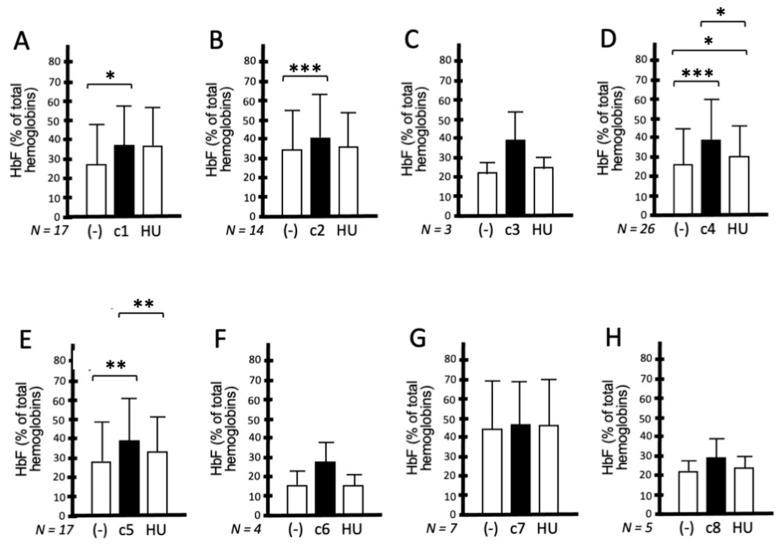
Increase in HbF production in ErPCs from β-thalassemia patients. ErPCs from different cohorts of patients were either untreated (-), treated with HU (200 nM), or treated with the isoxazole compounds **C1**–**C8** (**A**–**H**) (120 nM). Results represent the average ± S.D. (The number of independent ErPC cultures is indicated in each panel). * = *p* < 0.05 (significant); **, *** = *p* < 0.01 (highly significant).

Figure 5 shows representative HPLC analyses of the pattern of hemoglobin accumulation following treatment of ErPCs from a β^0^39/β^+^ISVI-110 patient, with **c1** and **c2** used at 200 nM concentration and **c4** used at 85, 120, 170, and 200 nM concentrations. The obtained results demonstrate that all the analyzed isoxazoles induce a preferential increase in HbF to an extent similar to, or even higher than, HU. The quantitative analysis is shown in Figure 6, which reports the data obtained using ErPCs from different cohorts of patients; these ErPCs were from the same cohort and were either untreated (-), treated with HU (200 nM), or treated with the isoxazole compounds **c1**–**c8** (120 nM).

**Figure 7:**
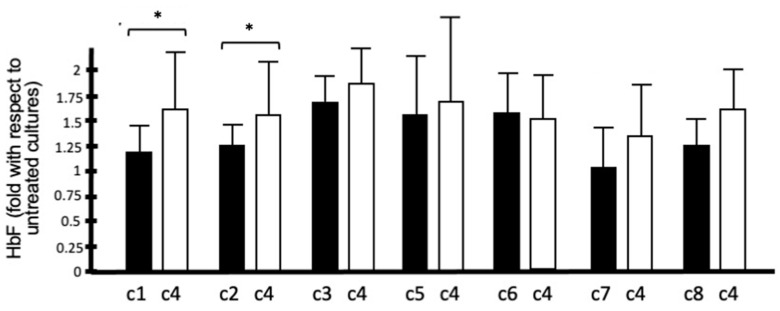
Induction of HbF production in ErPCs from β-thalassemia patients: activity of **c4** in comparison with the other isoxazole compounds. Results represent the average ± S.D. of at least three independent ErPC cultures. * = *p* < 0.05 (significant).

According to the flow chart depicted in Figure 4, we selected the best four compounds on the basis of their differences in HbF production with respect to untreated cells and cells treated with HU. The most active isoxazoles were compounds **c3**, **c4**, **c5,** and **c6**. All these compounds (unlike **c1**, **c2**, **c7,** and **c8**) exhibited high hemoglobin production with respect to untreated cells and HU-treated cells. In order to allow for meaningful comparisons, in all the experiments shown in Figure 6, the same cohort of β-thalassemia patients was treated with the isoxazole derivatives and HU. When the HbF-inducing activity of **c4** was compared to that exhibited by **c1**, **c2**, **c3**, **c5**, **c6**, **c7,** and **c8** (Figure 7), this compound was found to be more active (only **c6** exhibited an activity similar to **c4** in inducing HbF), suggesting that **c4** should be considered of interest for further studies.

### 2.4. Effects of 3,4-Isoxazolediamine Derivatives on Human Erythroid Precursor Cells: Isoxazole Derivatives Induce a Decrease in the Excess of Free α-Globins

**Figure 8:**
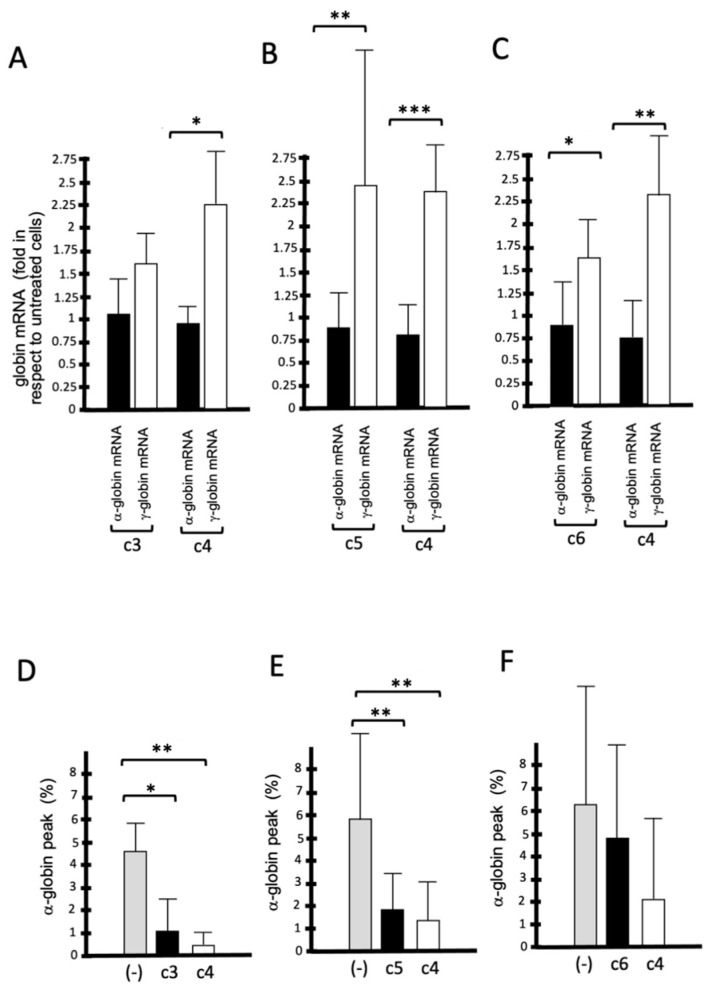
Preferential induction of γ-globin mRNA production (**A**–**C**) and reduction in the excess of free α-globin chains (**D**–**F**) in ErPCs from β-thalassemia patients treated with isoxazoles derivatives: activity of **c4** in comparison with **c3**, **c5,** and **c6**. Results represent the average ± S.D. of at least three independent experiments. * = *p* < 0.05 (significant); **, *** = *p* < 0.01 (highly significant).

All the isoxazoles compounds with potential in inducing an increase in HbF production by treated ErPCs (**c3**, **c4**, **c5,** and **c6**, see Figure 6) were also assayed for their possible effects in reducing the excess of α-globin. According to the interest in **c4**, the activity of this compound was assayed with the other bioactive isoxazoles **c3**, **c5,** and **c6**. Figure 8A shows that all the **c3**, **c4**, **c5,** and **c6** isoxazole compounds retain the ability to induce preferential accumulation of γ-globin mRNA by treated ErPCs from β-thalassemia patients. This was found to be highly significant.

Figure 8 also indicates that all the studied isoxazole compounds are very powerful in reducing the excess of free α-globin. In particular, the effects of **c4** were remarkable since this compound exhibited the highest activity in reducing the excess of free α-globin (Figure 8D–F). Following the scheme of this study depicted in Figure 4, **c4** was selected to further characterize its activity.

### 2.5. Compound c4 Is One of the Most Interesting 3,4-Isoxazolediamine Derivatives Studied: Characterization of Its Activity in Comparison with HbF Inducers Employed in Clinical Trials

**Figure 9:**
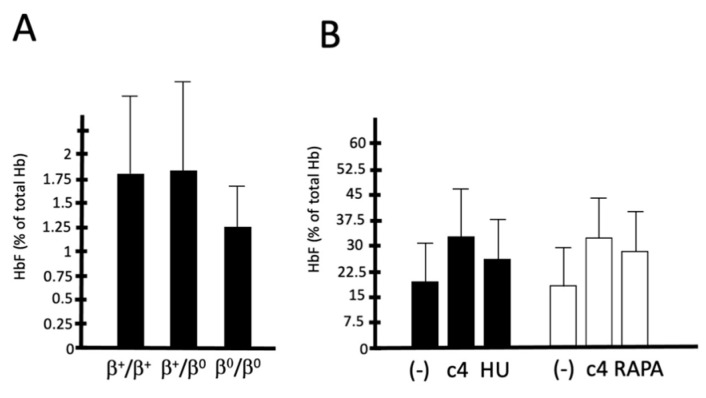
Induction HbF production by **c4** in ErPCs from β-thalassemia patients of different genotypes (β^+^/β^+^, β^0^/β^+^ and β^0^/β^0^) (**A**) and comparison with hydroxyurea (HU) and rapamycin (RAPA) (**B**). In panel (**A**,**B**) results are expressed as % of total hemoglobins analyzed by HPLC.

In order to verify the possible impact of **c4** in the development of therapeutic approaches for β-thalassemia, we examined the HbF inducing activity of **c4** (a) in ErPCs from patients with different genotypes (Figure 9A) and (b) in comparison with inducers presently employed in clinical trials (Figure 9B). We selected hydroxyurea and rapamycin; hydroxyurea is employed in several clinical trials, such as NCT03183375 and NCT00809042 [38,39,40,41,42,43], while rapamycin (sirolimus) is employed in two clinical trials (NCT03877809 (A Personalized Medicine Approach for β-thalassemia Transfusion Dependent Patients: Testing sirolimus in a First Pilot Clinical Trial) and NCT04247750 (Treatment of β-thalassemia Patients with Rapamycin (Sirolimus): From Pre-clinical Research to a Clinical Trial) [44].

Figure 9A shows that the effects of **c4** in inducing HbF are not dependent of the phenotype/genotype. The lower fold induction values found in ErPCs from β^0^/β^0^ patients may be due to the fact that those patients exhibit higher (and heterogenous) levels of endogenous HbF with respect to β^+^/β^+^ and β^+^/β^0^ patients. Figure 9B shows that c4 ability to induce HbF is similar to, or even higher in some ErPC cultures, than that exhibited by hydroxyurea and rapamycin.

**Figure 10:**
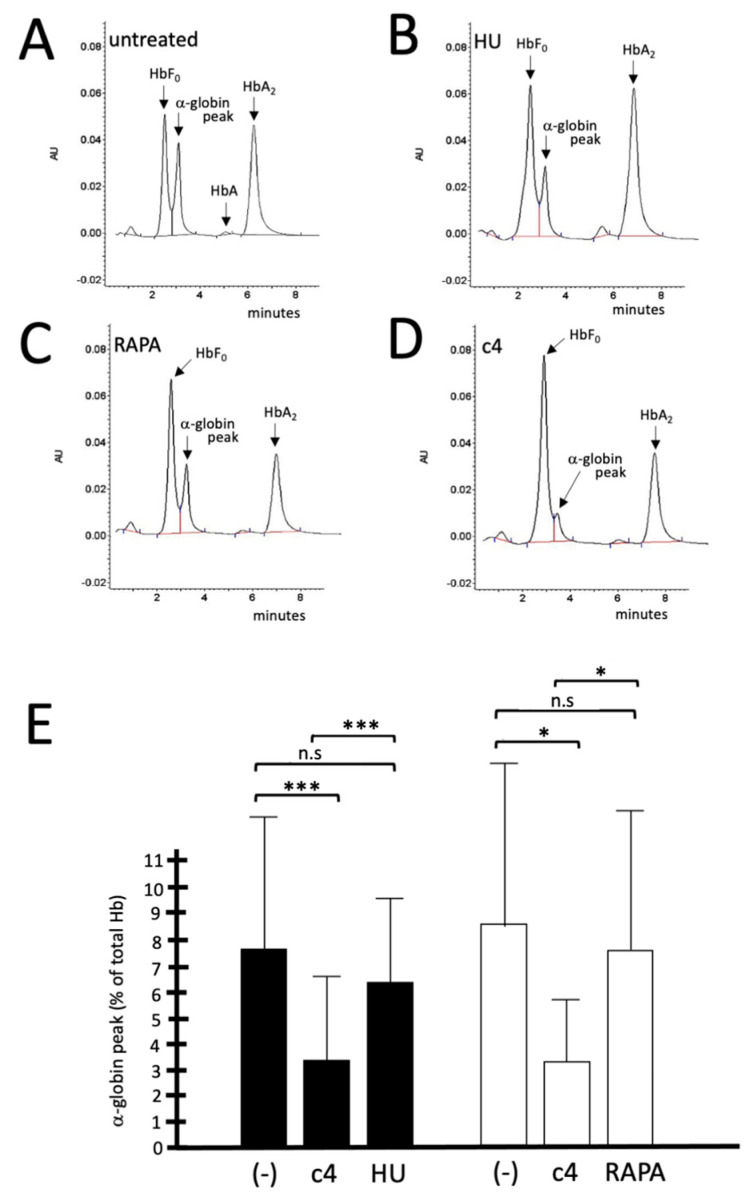
Reduction in the excess of free α-globin chains in ErPCs from β-thalassemia patients: activity of **c4** in comparison with that hydroxyurea (HU) and rapamycin (RAPA). (**A**–**D**): representative HPLC analyses performed on ErPC samples isolated from a β^0^/β^0^ thalassemia patients, either untreated (**A**) or treated with HU (**B**), rapamycin (**C**), and c4 (**D**); in panel (**E**) the results indicate the ratios **c4**/untreated, HU/untreated, and RAPA/untreated, as indicated (average ± S.D. of at least three independent experiments). * = *p* < 0.05 (significant); *** = *p* < 0.01 (highly significant); n.s.: not significant.

Finally, Figure 10 indicates that **c4** is more active than hydroxyurea and rapamycin in decreasing the accumulation of excess free α-globin chains. Representative HPLC analyses are shown in Figure 10A–D, while the results of at least four independent experiments are shown in Figure 10E, indicating that **c4** is clearly more efficient than HU and RAPA in reducing the free α-globin content. This is of great interest, as a reduction in the α-globin excess in erythroid cells from β-thalassemia patients is expected to be highly beneficial.

## 3. Discussion

The Hereditary Persistence of Fetal Hemoglobin (HPFH) phenotype is characterized by continuous HbF synthesis in the adult stage of erythropoiesis. The increased levels of fetal hemoglobin can improve the clinical course of β-thalassemia [7–11->7–10]. With the objective of mimicking this condition, extensive research activity on HbF inducers has been undertaken in order to find molecules of possible therapeutic interest [9].

Currently, hydroxyurea (HU) is the only one approved drug able to induce fetal hemoglobin [14,18,38,39,40,41]. However, the side effects (as leukopenia and neutropenia) of the HU treatment, and the fact that it is active only in some patients, attracts limited enthusiasm for the use of this molecule [42,43]. For these reasons, several research teams are trying to identify new molecules capable of inducing HbF expression with greater efficiency and lower toxicity than the HU [39].

The first aim of our study was to analyze eight isoxazole derivatives in order to find the best compound(s) for HbF induction. We excluded genotoxic effects (Supplementary Tables S1–S10). The first set of experiments were performed on K562 cells, confirming that all compounds were found to stimulate erythroid differentiation as well as an increase in fetal and embryonic globin mRNA content (Figure 2).

After this first screening, primary erythroid precursor cells (ErPCs) derived from 29 β-thalassemia patients were employed. These cells, isolated from patients with different genotypes (Figure 3), represent an excellent model system for screening HbF inducers that are to be considered in pre-clinical studies for developing therapeutic protocols for β-thalassemia. For this reason, in our study, the biological effects of the isoxazole derivatives were compared with those of two well-known HbF inducers, HU and rapamycin, which are both employed in clinical trials for β-thalassemia [37,38,39,40,41,42,43,44].

A second important end-point of our study was the demonstration that the isoxazole derivative treatment is associated with a decrease in the excess of α-globin produced by ErPCs. This is of relevance for the treatment of β-thalassemia, due to the unbalanced α-globin/β-globin ratio and the relative excess of free α-globin chains [11,12]. With respect to this point, there is a lot of evidence sustaining that the excess of free α-globin chains has a great impact on the pathophysiology of β-thalassemia [1,2,3]. Accordingly, the sharp effect of the isoxazole compounds on the excess of free α-globin chains produced by the treated ErPCs is relevant.

In our experiments, **c4** was found to be highly efficient in inducing HbF, in inducing γ-globin gene expression, and in reducing free α-globin chains. We should point out that, in our study, structure–activity relationship (SAR) analysis was not considered, at least for the following reasons: (a) low number of the compounds tested, that have been designed to identify the best compound(s) to be used for SAR studies; (b) heterogeneity of the effects examined (HbF production, reduction in the excess of free α-globin), most of which are under the control of complex molecular pathways; (c) heterogeneity of the phenotypes of the recruited β-thalassemia patients. SAR studies are, however, warranted to rationally develop even more active compounds. Nevertheless, compound **c4** deserves, in our opinion, further research efforts for determining its real impact for pre-clinical studies aimed at developing possible therapeutic treatments for β-thalassemia.

## 4. Materials and Methods

### 4.1. Chemical Compounds

The synthesis of the isoxazole derivatives and their preliminary characterization as inducers of erythroid differentiation and hemoglobin production by human leukemic K562 cells has been previously reported [31]. All the compounds were dissolved in DMSO and further dilutions were made with ethanol (EtOH). The stock solutions were stored at −20 °C and thawed just before the treatment.

### 4.2. Cell Cultures

#### 4.2.1. Human Erythroleukemic K562 Cells

The cells were isolated and characterized by Lozzio CB and Lozzio BB, from a patient with chronic myelogenous leukemia (CML) in blast crisis [32,33]. K562 cells were cultured in humidified atmosphere of 5% CO_2_ in RPMI-1640 medium (Lonza, Verviers, Belgium) supplemented with 10% fetal bovine serum (FBS; Biowest, Nuaillé, France), 50 units/mL penicillin (Lonza, Verviers, Belgium), and 50 μg/mL streptomycin (Lonza, Verviers, Belgium) [33].

#### 4.2.2. Human Erythroid Precursors Cells (ErPCs)

The two-phase liquid culture procedure has been previously described [16,36,37]. Following informed consent, peripheral blood samples from healthy donors and β-thalassemia patients were isolated. The institutional review boards of Ferrara University approved this study. Mononuclear cells were isolated by Lympholyte-H density gradient centrifugation (Nycograde^TM^ polysucrose 400 and sodium diatrizoate Celbio, Milano, Italy), washed three times in 1X PBS solution, and cultured for 7 days in α-medium (Sigma-Aldrich, St. Louis, MI, USA) supplemented with 10% FCS (GIBCO, BRL, Life Technologies, Milano, Italy). In addition, we used 1 μg/mL cyclosporine A (Sigma-Aldrich, St. Louis, MI, USA), and 10% conditioned medium from the 5637 bladder carcinoma cell line [16]. The cultures were incubated at 37 °C under an atmosphere of 5% CO_2_ in air with extra humidity. After the first culture phase, the non-adherent cells were harvested, washed, and cultured in α-medium with 30% FCS, 1% deionized bovine serum albumin (Sigma-Aldrich, St. Louis, MI, USA), 10–5 M β-mercaptoethanol, 2 mM L-glutamine, 10–6 M dexamethasone, 1 U/mL human recombinant erythropoietin (Tebu-bio, Magenta, Italy), and 10 ng/mL stem cell factor (SCF, PeproTech EC Ltd., London, England). After the second culture phase (7 days long), the compounds to be tested were added and the treatment carried out for a further 5 days. The cellular samples were centrifuged, washed, and employed for real time RT-PCR reactions and HPLC analysis.

### 4.3. Cell Proliferation Analysis and Erythroid Differentiation on K562 Cell Line

Human leukemia K562 cells were seeded at 30,000/mL and the treatments were carried out by adding the appropriate drug concentration. The cell number/mL was analyzed using a model Z2 Coulter counter (Coulter Electronics, Hialeah, FL, USA) after day 2 and day 3 in order to determine any possible effects on cell proliferation, and the IC50 value of each compound (the concentration causing 50% growth inhibition) was determined. Erythroid differentiation was assayed by counting benzidine/H_2_O_2_ positive cells in a solution containing 0.2% benzidine in 5 M glacial acetic acid and 10% H_2_O_2_, as described [31].

### 4.4. RNA Extraction from K562 Cells and ErPCs

The cells were isolated by centrifugation at 1200× *g* for 10 min at 4 °C after washing in 1X PBS and lysed in Tri-reagent^TM^ (Sigma-Aldrich, St. Louis, MI, USA). The homogenate was incubated for 5 min at room temperature, added to 0.2 mL of chloroform per ml of Tri-reagent^TM^, vigorously shaken for 15 s, incubated 5 min at room temperature, and finally centrifuged at 14,000× *g* for 15 min at 4 °C. The aqueous phase was removed and added to 0.5 mL of isopropanol per ml of Tri-reagent^TM^. After 10 min at room temperature, the samples were centrifuged at 14,000× *g* for 15 min at 4 °C. The RNA pellets were washed with 1 mL of 75% ethanol and centrifuged at 14,000× *g* for 5 min at 4 °C. Finally, the pellets were suspended in RNase-free water to be analyzed on 1% agarose gel.

### 4.5. RT-PCR Analysis

For the synthesis of cDNA with random hexamers (TaqMan^®^ Reverse TranscriptionReagents, from Life Technologies, Carlsbad, CA, USA), 500 μg of total RNA were used. A quantitative real-time PCR assay was carried out using gene-specific double fluorescently labelled probes in a 7700 Sequence Detection System version 1.7 (Life Technologies, Carlsbad, CA, USA) [37] The nucleotide sequences used for real-time qPCR analysis of α-, β-, and γ-globin mRNAs are reported here: α-globin forward primer, 5′-CAC GCG CAC AAG CTT CG-3′; α-globin reverse primer, 5′-AGG GTC ACC AGC AGG CAG T-3′; α-globin probe, 5′-FAM-TGG ACC CGG TCA ACT TCA AGC TCC T-TAMRA-3′; β-globin forward primer, 5′-CAA GAA AGT GCT CGG TGC CT-3′; β-globin reverse primer, 5′-GCA AAG GTG CCC TTG AGG T-3′; β-globin probe, 5′-FAM-TAG TGA TGG CCT GGC TCA CCT GGA C-TAMRA-3′; γ-globin forward primer, 5′-TGG CAA GAA GGT GCT GAC TTC-3′; γ-globin reverse primer, 5′-TCA CTC AGC TGG GCA AAG G-3′; γ-globin probe, 5′-FAM-TGG GAG ATG CCA TAA AGC ACC TGG-TAMRA-3′ [16,19]. The fluorescent reporter and the quencher were: 6 carboxyfluorescein (FAM) and 6-carboxy-N,N,N′,N′-tetramethylrhodamine (TAMRA), respectively. Human glyceraldehyde-3-phosphate dehydrogenase (GAPDH) and β-actin fluorescently labelled with VIC™ (Life Technologies, Applied Biosystems, Foster City, CA, USA) were used as reference genes [37].

### 4.6. HPLC Analysis

Human erythroid precursor cells were harvested, counted (Z2 Coulter Counter, Coulter Electronics, Hialeah, FL, USA), and washed once with PBS. Then, the pellets were lysed in H_2_O. After incubation on ice for 20 min and centrifugation for 5 min at 14,000 rpm in a microcentrifuge, the supernatant was separated from the membrane debris and injected (20 μL). Hb proteins present in the lysates were separated by cation-exchange HPLC, utilizing a Beckman Coulter instrument System Gold 126 Solvent Module-166 Detector. Hemoglobin was separated using a PolyCAT-A column, samples were eluted in a solvent gradient utilizing aqueous sodium chloride–BisTris–KCN buffers, and detection was performed at 415 nm. The standard controls were the purified HbA (SIGMA, St Louis, MO, USA) and HbF (Alpha Wassermann, Milano, Italy) [37].

### 4.7. Analysis of Genotoxicity

A mutagenicity assay (Ames test) was performed for the compounds 1–8 and HU, following the plate incorporation method with the histidine-requiring Salmonella typhimurium mutant TA97A, TA98, TA100, and TA1535 strains purchased from Molecular Toxicology Inc. MOLTOX (Boone, NC, USA). All strains (100 µL per plate of fresh overnight cultures) were checked with and without the addition of 0.5 mL of a 5% S9 exogenous metabolic activator (S9 mix). The lyophilized post-mitochondrial supernatant S9 mix (Aroclor 1254-induced, Sprague–Dawley male rat liver in 0.154 M KCl solution), commonly used for the activation of pro-mutagens to mutagenic metabolites (Molecular Toxicology, Inc., Boone, NC, USA) was stored at −80 °C before use. The concentrations tested for all the samples were 1, 5, 10, 50, and 100 µg/plate (Supplementary Materials). Sodium azide, 1 μg/plate, was used as a positive mutagenic control.

### 4.8. Statistical Analysis

The data were normally distributed and presented as mean ± S.D. Statistical differences between groups were compared using the Prism Software v9.02 and the two-tail paired *t*-test. Statistical differences were considered significant when *p* < 0.05 (*), highly significant when *p* < 0.01 (**).

## 5. Conclusions

The novel isoxazole derivatives studied here are potent and non-toxic inducers of HbF production. Their activity approaches (and in some case is higher than) the activity of two HbF inducers that are at present under clinical trials (hydroxyurea and sirolimus). Among the analyzed isoxazole derivatives, compound c4 exhibited high-HbF-induction activity and high efficiency in the reduction in the excess of free α-globin chains. For these reasons, compound c4 can be considered a strong candidate for ameliorating the phenotype of β-thalassemia, reducing also ineffective erythropoiesis. Accordingly, compound c4 deserves further experimental activity for determining its real impact for pre-clinical studies aimed at developing possible therapeutic treatments for β-thalassemia.

Further investigations, in our opinion, should include pharmacogenomics studies, transcriptomic analyses, and an extensive analysis of the possible correlation between genotype (including DNA polymorphisms) and HbF induction. Our analysis, performed using cells from 29 different β-thalassemia patients, suggests the possibility of also identifying subjects that might be further selected for a clinical trial because they respond well to the ex vivo treatment with the active isoxazole derivatives.

## 6. Patents

Related to this study is the United States Patent 11,077,116 (isoxazole derivatives as inducers of fetal hemoglobin in erythroid precursor cells from beta-thalassemic patients). The invention refers to the use of isoxazole derivatives to prepare medicament able to induce fetal hemoglobin (HbF) synthesis in β-thalassemia and sickle cell disease (SCD) patients.

## Data Availability

Additional information and data will be freely available upon request to the correspondence authors.

## Supplementary Materials

The following supporting information can be downloaded at: https://www.mdpi.com/article/10.3390/molecules29010008/s1, Tables S1–S10: Genotoxicity of the studied isoxazole analogues.
