# Supplementary material for: New Synthetic Isoxazole Derivatives Acting as Potent Inducers of Fetal Hemoglobin in Erythroid Precursor Cells Isolated from β-Thalassemic Patients
Source: Molecules. 2023 Dec 19;29(1):8. doi: 10.3390/molecules29010008 (PMC10779815; doi:10.3390/molecules29010008)
Supplement: Supplementary file 1 [file molecules-29-00008-s001.zip › molecules-2745646-Supplementary.pdf]

molecules

MDPI

Article

New Synthetic Isoxazole Derivatives acting as potent Inducers of Fetal Hemoglobin in Erythroid Precursor Cells isolated from β-Thalassemic Patients

Cristina Zuccato[1,2], Lucia Carmela Cosenza [1], Chiara Tupini,[1] Alessia Finotti,[1] Gianni Sacchetti,[1] Daniele Simoni,[3] Roberto Gambari[1,2,*] and Ilaria Lampronti [1,2*]

[1] Department of Life Sciences and Biotechnology, Section of Biochemistry and Molecular Biology, Ferrara University, 44121 Ferrara, Italy; luciacarmela.cosenza@unife.it (L.C.C.); cristina.zuccato@unife.it (C.Z.); alessia.finotti@unife.it (A.F.); chiara.tupini@unife.it (C.T.)

[2] Center "Chiara Gemmo and Elio Zago" for the Research on Thalassemia, University of Ferrara, 44121 Ferrara, Italy; gam@unife.it (R.G.); alessia.finotti@unife.it (A.F.); ilaria.lampronti@unife.it (I.L.); cristina.zuccato@unife.it (C.Z.)

[3] Department of Chemical, Pharmaceutical and Agricultural sciences, Ferrara University, 44121 Ferrara, Italy; daniele.simoni@unife.it (D.S.)

* Correspondence: ilaria.lampronti@unife.it (I.L.) Tel.: +390532-974509; gam@unife.it (R.G.) Tel.: +39-0532-974443

Supplementary material

HU

	Salmonella typhimurium **TA97A**						*Salmonella typhimurium* **TA98**						*Salmonella typhimurium* **TA100**						*Salmonella typhimurium* **1535**					
	**- S9**			**+ S9**			**- S9**			**+ S9**			**- S9**			**+ S9**			**- S9**			**+ S9**		
	**average**	**SD**	**t/c**	**average**	**SD**	**t/c**	**average**	**SD**	**t/c**	**average**	**SD**	**t/c**	**average**	**SD**	**t/c**	**average**	**SD**	**t/c**	**average**	**SD**	**t/c**	**avera ge**	**SD**	**t/c**
**DMSO**	108.0	8.9	1.0	115.0	14.3	1.0	93.5	2.5	1.0	79.2	4.0	1.0	109.2	15.4	1.0	43.4	3.2	1.0	58.8	4.8	1.0	58.0	11.8	1.0
**C+**	351.0	35.8	3.3	559.0	44.8	4.9	1054.7	74.4	11.3	331.0	40.6	4.2	478.4	26.9	4.4	285.0	5.4	2.9	220.1	3.0	3.9	191.7	5.3	3.3
**1**	114.0	5.2	1.1	93.0	6.9	0.8	88.0	3.8	0.9	118.8	28.6	1.5	88.2	12.8	0.8	97.1	9.8	1.0	84.0	2.6	1.4	89.8	5.1	1.5
**5**	116.0	20.0	1.1	80.5	11.4	0.7	78.1	3.8	0.8	106.7	13.3	1.3	90.3	11.1	0.8	109.7	21.2	1.1	76.7	6.8	1.3	**114.7**	**13.3**	**2.0**
**10**	59.0	6.9	0.5	68.0	0.9	0.6	93.5	7.6	1.0	89.1	5.7	1.1	99.4	17.0	0.9	87.7	5.4	0.9	89.5	10.0	1.5	109.3	2.3	1.9
**50**	98.0	49.5	0.9	98.0	7.5	0.9	95.7	15.1	1.0	123.2	10.1	1.6	122.5	11.6	1.1	134.7	11.8	1.4	73.7	4.6	1.3	73.5	14.5	1.3
**100**	96.0	3.0	0.9	62.0	3.8	0.5	78.1	6.9	0.8	127.6	6.9	1.6	116.9	2.4	1.1	153.5	33.3	1.6	59.0	11.3	1.0	54.5	8.3	0.9

Table S1. Lack of genotoxic activity of Hydroxyurea (HU) at different concentrations (1, 5, 10, 50, 100 μg/plate). All the t/c values were much lower than those obtained using a positive control (sodium azide, 1 μg/plate).

molecules

MDPI

C1

	Salmonella typhimurium **TA97A**						*Salmonella typhimurium* **TA98**						*Salmonella typhimurium* **TA100**						*Salmonella typhimurium* **1535**					
	**- S9**			**+ S9**			**- S9**			**+ S9**			**- S9**			**+ S9**			**- S9**			**+ S9**		
	**average**	**SD**	**t/c**	**average**	**SD**	**t/c**	**average**	**SD**	**t/c**	**average**	**SD**	**t/c**	**average**	**SD**	**t/c**	**average**	**SD**	**t/c**	**average**	**SD**	**t/c**	**average**	**SD**	**t/c**
**DMSO**	108.0	8.9	1.0	138.0	9.5	1.0	93.5	8.3	1.0	79.2	13.2	1.0	109.2	15.4	1.0	97.1	3.2	1.0	58.8	4.8	1.0	58.0	11.8	1.0
**C+**	351.0	35.8	3.3	559.0	44.8	4.0	1054.7	74.4	10.0	331.0	40.6	4.2	478.4	26.9	4.4	285.0	5.4	2.9	220.1	3.0	3.9	191.7	5.3	3.3
**1**	68.1	27.2	0.6	151.2	14.7	1.1	67.1	1.9	0.7	83.6	5.0	1.1	132.3	4.2	1.2	126.9	16.3	1.3	42.5	0.0	0.7	52.7	7.2	0.9
**5**	84.6	9.0	0.8	165.0	22.4	1.2	53.9	1.9	0.6	82.5	0.0	1.0	135.1	2.4	1.2	111.2	10.9	1.1	30.8	3.2	0.5	74.0	1.0	1.3
**10**	83.7	17.6	0.8	157.8	3.7	1.1	70.4	3.8	0.8	83.6	1.9	1.1	146.3	17.1	1.3	125.3	15.1	1.3	31.7	1.4	0.5	82.3	2.1	1.4
**50**	76.3	16.5	0.7	145.8	4.8	1.1	62.7	11.4	0.7	67.1	19.3	0.8	122.5	5.3	1.1	103.4	18.8	1.1	50.3	2.0	0.9	83.3	9.8	1.4
**100**	66.4	6.0	0.6	132.6	15.5	1.0	53.9	15.6	0.6	63.8	3.8	0.8	123.2	17.5	1.1	139.4	5.4	1.4	39.8	1.0	0.7	70.8	1.0	1.2

Table S2. Lack of genotoxic activity of **c1** at different concentrations (1, 5, 10, 50, 100 µg/plate). All the t/c values were much lower than those obtained using a positive control (sodium azide, 1 µg/plate).

C2

	Salmonella typhimurium **TA97A**						*Salmonella typhimurium* **TA98**						*Salmonella typhimurium* **TA100**						*Salmonella typhimurium* **1535**					
	**- S9**			**+ S9**			**- S9**			**+ S9**			**- S9**			**+ S9**			**- S9**			**+ S9**		
	**average**	**SD**	**t/c**	**average**	**SD**	**t/c**	**average**	**SD**	**t/c**	**average**	**SD**	**t/c**	**average**	**SD**	**t/c**	**average**	**SD**	**t/c**	**average**	**SD**	**t/c**	**average**	**SD**	**t/c**
**DMSO**	102.0	6.0	1.0	109.0	6.9	1.0	105.2	4.0	1.0	137.4	4.7	1.0	109.2	15.4	1.0	97.1	15.1	1.0	58.8	4.8	1.0	58.0	11.8	1.0
**C+**	351.0	35.8	3.4	559.0	44.8	5.1	1054.7	74.4	10.0	331.0	40.6	2.4	478.4	26.9	4.4	285.0	5.4	2.9	220.1	3.0	3.9	191.7	5.3	3.3
**1**	90.2	5.2	0.9	112.1	32.9	1.0	143.5	16.4	1.4	125.0	1.8	0.9	115.5	3.6	1.1	87.7	7.2	0.9	31.0	4.1	0.5	51.7	2.9	0.9
**5**	62.0	4.6	0.6	99.0	37.5	0.9	97.0	43.5	0.9	146.7	10.9	1.1	153.3	15.9	1.4	87.7	17.8	0.9	37.8	0.6	0.6	61.7	9.5	1.1
**10**	90.3	9.0	0.9	125.5	14.2	1.1	143.5	0.0	1.4	130.2	6.2	0.9	134.4	23.4	1.2	78.3	26.7	0.8	37.2	0.3	0.6	63.0	0.0	1.1
**50**	70.1	4.6	0.7	149.4	20.0	1.4	139.4	14.2	1.3	138.5	22.4	1.0	141.4	2.4	1.3	72.1	16.5	0.7	39.5	3.0	0.7	76.3	5.5	1.3
**100**	66.0	13.7	0.6	141.0	7.9	1.3	**214.6**	**22.6**	**2.0**	106.4	3.6	0.8	144.9	17.2	1.3	70.5	12.4	0.7	21.7	4.0	0.4	57.0	6.6	1.0

Table S3. Lack of genotoxic activity of **c2** at different concentrations (1, 5, 10, 50, 100 µg/plate). All the t/c values were much lower than those obtained using a positive control (sodium azide, 1 µg/plate).

molecules

MDPI

C3

	Salmonella typhimurium **TA97A**						*Salmonella typhimurium* **TA98**						*Salmonella typhimurium* **TA100**						*Salmonella typhimurium* **1535**					
	**- S9**			**+ S9**			**- S9**			**+ S9**			**- S9**			**+ S9**			**- S9**			**+ S9**		
	**average**	**SD**	**t/c**	**average**	**SD**	**t/c**	**average**	**SD**	**t/c**	**average**	**SD**	**t/c**	**average**	**SD**	**t/c**	**average**	**SD**	**t/c**	**average**	**SD**	**t/c**	**avera ge**	**SD**	**t/c**
**DMSO**	102.0	6.0	1.0	109.0	6.9	1.0	105.2	4.0	1.0	137.4	4.7	1.0	109.2	15.4	1.0	97.1	15.1	1.0	58.8	4.8	1.0	58.0	11.8	1.0
**C+**	351.0	35.8	3.4	559.0	44.8	5.1	1054.7	74.4	10.0	331.0	40.6	2.4	478.4	26.9	4.4	285.0	5.4	2.9	220.1	3.0	3.9	191.7	5.3	3.3
**1**	190.0	17.1	1.8	85.4	18.3	0.8	121.6	17.1	1.2	150.9	7.8	1.1	85.4	2.4	0.8	84.6	12.4	0.9	53.7	1.2	0.9	40.7	1.5	0.7
**5**	197.2	22.7	1.9	88.0	13.9	0.8	108.0	12.5	1.0	132.3	7.8	1.0	137.9	9.5	1.3	81.5	9.8	0.9	41.7	1.4	0.7	66.7	9.2	1.1
**10**	173.6	13.9	1.7	102.6	15.9	0.9	110.7	18.8	1.1	139.5	37.6	1.0	135.1	2.4	1.2	90.9	28.7	1.0	37.3	4.6	0.6	51.0	5.3	0.9
**50**	170.6	17.6	1.7	104.2	6.9	0.9	132.6	10.3	1.3	124.0	3.1	0.9	138.6	5.6	1.3	103.4	12.4	1.1	29.5	2.3	0.5	57.3	0.6	1.0
**100**	166.5	32.9	1.6	82.2	1.7	0.8	102.5	8.2	1.0	132.3	1.8	1.0	137.2	1.2	1.3	70.5	12.4	0.7	49.8	0.6	0.8	52.0	5.6	0.9

Table S4. Lack of genotoxic activity of **c3** at different concentrations (1, 5, 10, 50, 100 µg/plate). All the t/c values were much lower than those obtained using a positive control (sodium azide, 1 µg/plate).

C4

	Salmonella typhimurium **TA97A**						*Salmonella typhimurium* **TA98**						*Salmonella typhimurium* **TA100**						*Salmonella typhimurium* **1535**					
	**- S9**			**+ S9**			**- S9**			**+ S9**			**- S9**			**+ S9**			**- S9**			**+ S9**		
	**average**	**SD**	**t/c**	**average**	**SD**	**t/c**	**average**	**SD**	**t/c**	**average**	**SD**	**t/c**	**average**	**SD**	**t/c**	**average**	**SD**	**t/c**	**average**	**SD**	**t/c**	**avera ge**	**SD**	**t/c**
**DMSO**	108.0	8.9	1.0	138.0	9.5	1.0	93.5	8.3	1.0	79.2	13.2	1.0	109.2	15.4	1.0	97.1	3.2	1.0	58.8	4.8	1.0	58.0	11.8	1.0
**C+**	351.0	35.8	3.3	559.0	44.8	4.0	1054.7	74.4	11.3	331.0	40.6	4.2	478.4	26.9	4.4	285.0	5.4	2.9	220.1	3.0	3.9	191.7	5.3	3.3
**1**	100.3	20.4	0.9	129.0	14.5	0.9	63.8	3.8	0.7	73.7	3.8	0.9	97.3	2.4	0.9	120.6	19.6	1.2	49.5	5.5	0.8	75.8	14.1	1.3
**5**	108.7	18.0	1.0	144.0	8.2	1.0	57.2	15.2	0.6	68.2	21.0	0.9	91.0	1.2	0.8	119.1	17.8	1.2	70.8	14.9	1.2	41.2	4.3	0.7
**10**	97.0	14.2	0.9	148.2	5.8	1.1	50.6	24.8	0.5	135.3	34.9	1.7	104.3	17.6	1.0	139.4	2.7	1.4	64.0	10.5	1.1	48.0	5.2	0.8
**50**	82.6	22.5	0.8	151.2	26.5	1.1	45.1	3.8	0.5	115.5	5.7	1.5	109.9	11.6	1.0	158.2	11.8	1.6	73.5	0.5	1.2	51.0	8.2	0.9
**100**	83.1	20.4	0.8	138.6	12.5	1.0	40.7	10.6	0.4	119.9	3.8	1.5	105.0	8.4	1.0	115.9	17.8	1.2	77.2	13.1	1.3	48.2	8.0	0.8

Table S5. Lack of genotoxic activity of **c4** at different concentrations (1, 5, 10, 50, 100 µg/plate). All the t/c values were much lower than those obtained using a positive control (sodium azide, 1 µg/plate).

molecules

MDPI

C5

	Salmonella typhimurium **TA97A**						*Salmonella typhimurium* **TA98**						*Salmonella typhimurium* **TA100**						*Salmonella typhimurium* **1535**					
	**- S9**			**+ S9**			**- S9**			**+ S9**			**- S9**			**+ S9**			**- S9**			**+ S9**		
	**average**	**SD**	**t/c**	**average**	**SD**	**t/c**	**average**	**SD**	**t/c**	**average**	**SD**	**t/c**	**average**	**SD**	**t/c**	**average**	**SD**	**t/c**	**average**	**SD**	**t/c**	**average**	**SD**	**t/c**
**DMSO**	102.0	6.0	1.0	109.0	6.9	1.0	105.2	4.0	1.0	137.4	4.7	1.0	109.2	15.4	1.0	97.1	15.1	1.0	58.8	4.8	1.0	58.0	11.8	1.0
**C+**	351.0	35.8	3.4	559.0	44.8	5.1	1054.7	74.4	10.0	331.0	40.6	2.4	478.4	26.9	4.4	285.0	5.4	2.9	220.1	3.0	3.9	191.7	5.3	3.3
**1**	109.1	36.7	1.1	155.1	3.5	1.4	132.6	8.5	1.3	126.1	10.9	0.9	135.8	3.2	1.2	73.6	5.4	0.8	47.3	1.8	0.8	54.2	1.6	0.9
**5**	87.9	6.9	0.9	151.2	11.4	1.4	129.8	9.5	1.2	139.5	16.1	1.0	122.5	3.2	1.1	109.7	17.8	1.1	47.3	2.3	0.8	62.7	5.9	1.1
**10**	86.5	1.7	0.8	177.4	20.8	1.6	121.6	9.5	1.2	107.5	4.7	0.8	123.9	7.3	1.1	98.7	9.4	1.0	51.3	6.8	0.9	55.5	2.6	1.0
**50**	76.0	1.7	0.7	133.0	7.5	1.2	120.3	22.6	1.1	151.9	0.0	1.1	126.7	15.8	1.2	70.5	0.0	0.7	64.5	11.8	1.1	50.3	1.4	0.9
**100**	92.4	1.7	0.9	90.3	6.0	0.8	131.2	7.1	1.2	156.0	14.3	1.1	113.4	5.6	1.0	78.3	2.7	0.8	38.2	0.6	0.6	57.7	3.0	1.0

Table S6. Lack of genotoxic activity of **c5** at different concentrations (1, 5, 10, 50, 100 µg/plate). All the t/c values were much lower than those obtained using a positive control (sodium azide, 1 µg/plate).

C6

	Salmonella typhimurium **TA97A**						*Salmonella typhimurium* **TA98**						*Salmonella typhimurium* **TA100**						*Salmonella typhimurium* **1535**					
	**- S9**			**+ S9**			**- S9**			**+ S9**			**- S9**			**+ S9**			**- S9**			**+ S9**		
	**average**	**SD**	**t/c**	**average**	**SD**	**t/c**	**average**	**SD**	**t/c**	**average**	**SD**	**t/c**	**average**	**SD**	**t/c**	**average**	**SD**	**t/c**	**average**	**SD**	**t/c**	**average**	**SD**	**t/c**
**DMSO**	102.0	6.6	1.0	144.5	17.8	1.0	90.3	7.6	1.0	82.0	7.0	1.0	112.7	6.0	1.0	136.9	14.8	1.0	55.8	6.5	1.0	65.8	10.4	1.0
**C+**	351.0	35.8	3.4	559.0	44.8	3.9	1054.7	74.4	11.7	331.0	40.6	4.0	478.4	26.9	4.2	285.0	5.4	2.1	220.1	3.0	3.9	191.7	5.3	2.9
**1**	74.0	18.3	0.7	165.0	5.4	1.2	65.8	16.8	0.7	86.7	3.1	1.1	127.4	6.1	1.1	107.3	6.4	0.8	67.3	0.6	1.2	77.2	16.3	1.2
**5**	133.1	3.1	1.3	192.2	11.3	1.3	83.3	8.7	0.9	81.0	11.1	1.0	115.5	3.6	1.0	148.0	6.4	1.1	65.5	3.5	1.2	73.0	2.0	1.1
**10**	179.3	9.0	1.8	157.1	25.7	1.2	75.6	12.8	0.8	75.0	2.0	0.9	138.6	3.6	1.2	169.0	28.0	1.2	85.0	14.7	1.5	73.8	6.8	1.1
**50**	152.8	14.0	1.5	128.0	16.5	0.9	73.5	16.4	0.8	66.3	0.6	0.8	144.9	7.6	1.3	231.9	20.4	1.7	65.2	2.0	1.2	67.7	1.2	1.0
**100**	99.3	14.7	1.0	90.3	8.4	0.6	100.1	4.4	1.1	19.7	4.9	0.2	146.3	4.8	1.3	169.0	29.9	1.2	70.3	9.1	1.3	78.3	4.6	1.2

Table S7. Lack of genotoxic activity of **c6** at different concentrations (1, 5, 10, 50, 100 µg/plate). All the t/c values were much lower than those obtained using a positive control (sodium azide, 1 µg/plate).

C7

	Salmonella typhimurium **TA97A**						*Salmonella typhimurium* **TA98**						*Salmonella typhimurium* **TA100**						*Salmonella typhimurium* **1535**					
	**- S9**			**+ S9**			**- S9**			**+ S9**			**- S9**			**+ S9**			**- S9**			**+ S9**		
	**average**	**SD**	**t/c**	**average**	**SD**	**t/c**	**average**	**SD**	**t/c**	**average**	**SD**	**t/c**	**average**	**SD**	**t/c**	**average**	**SD**	**t/c**	**average**	**SD**	**t/c**	**avera ge**	**SD**	**t/c**
**DMSO**	102,0	6,6	1,0	144,5	17,8	1,0	90.3	7.6	1.0	82.0	7.0	1.0	112.7	6.0	1.0	136.9	14.8	1.0	55.8	6.5	1.0	65.8	10.4	1.0
**C+**	351.0	35.8	3.4	559.0	44.8	3.9	1054.7	74.4	11.7	331.0	40.6	4.0	478.4	26.9	4.2	285.0	5.4	2.1	220.1	3.0	3.9	191.7	5.3	2.9
**1**	87,3	4,6	0,9	130,5	10,5	0,9	81.2	5.3	0.9	70.7	5.8	0.9	191.1	12.8	1.7	141.8	20.4	1.0	76.8	8.6	1.4	83.7	17.2	1.3
**5**	93,3	3,1	0,9	118,0	13,9	0,8	68.6	12.3	0.8	84.3	8.5	1.0	174.3	15.1	1.5	99.9	6.4	0.7	76.3	6.0	1.4	95.0	10.8	1.4
**10**	102,7	4,2	1,0	112,0	10,2	0,8	70.0	15.8	0.8	84.7	17.9	1.0	179.2	20.7	1.6	156.6	7.7	1.1	72.8	7.8	1.3	74.0	13.0	1.1
**50**	131,3	8,1	1,3	76,5	1,5	0,5	77.7	4.2	0.9	65.3	5.0	0.8	157.5	0.0	1.4	144.3	0.0	1.1	74.8	1.2	1.3	87.7	2.5	1.3
**100**	108,0	5,3	1,1	78,5	17,9	0,5	78.4	1.2	0.9	103.3	16.0	1.3	158.9	6.1	1.4	169.0	59.8	1.2	71.2	5.6	1.3	83.2	1.6	1.3

Table S8. Lack of genotoxic activity of **c7** at different concentrations (1, 5, 10, 50, 100 µg/plate). All the t/c values were much lower than those obtained using a positive control (sodium azide, 1 µg/plate).

C8

	Salmonella typhimurium **TA97A**						*Salmonella typhimurium* **TA98**						*Salmonella typhimurium* **TA100**						*Salmonella typhimurium* **1535**					
	**- S9**			**+ S9**			**- S9**			**+ S9**			**- S9**			**+ S9**			**- S9**			**+ S9**		
	**average**	**SD**	**t/c**	**average**	**SD**	**t/c**	**average**	**SD**	**t/c**	**average**	**SD**	**t/c**	**average**	**SD**	**t/c**	**average**	**SD**	**t/c**	**average**	**SD**	**t/c**	**avera ge**	**SD**	**t/c**
**DMSO**	102.0	6.6	1.0	136.7	15.0	1.0	90.3	7.6	1.0	82.0	7.0	1.0	112.7	6.0	1.0	136.9	14.8	1.0	55.8	6.5	1.0	65.8	10.4	1.0
**C+**	351.0	35.8	3.4	559.0	44.8	4.1	1054.7	74.4	11.7	331.0	40.6	4.0	478.4	26.9	4.2	285.0	5.4	2.1	220.1	3.0	3.9	191.7	5.3	2.9
**1**	84.7	5.8	0.8	168.2	39.3	1.2	58.8	5.6	0.7	73.3	2.9	0.9	98.7	9.2	0.9	119.6	22.3	0.9	60.3	9.5	1.1	71.8	15.1	1.1
**5**	77.3	1.2	0.8	141.2	11.1	1.0	68.6	16.0	0.8	72.0	0.0	0.9	101.5	3.2	0.9	129.5	3.7	0.9	61.7	1.2	1.1	60.7	4.5	0.9
**10**	71.2	4.6	0.7	127.7	6.7	0.9	156.8	1.2	1.7	44.3	4.0	0.5	98.7	18.7	0.9	161.6	11.9	1.2	72.3	1.2	1.3	72.2	11.6	1.1
**50**	72.0	3.5	0.7	127.3	19.2	0.9	107.1	7.3	1.2	83.3	19.4	1.0	106.4	10.4	0.9	152.9	18.3	1.1	47.3	0.6	0.8	61.7	10.4	0.9
**100**	103.7	7.0	1.0	124.3	5.5	0.9	81.9	4.2	0.9	86.7	9.3	1.1	140.7	2.1	1.2	319.4	20.4	2.3	50.2	2.9	0.9	72.8	0.3	1.1

Table S9. Lack of genotoxic activity of **c8** at different concentrations (1, 5, 10, 50, 100 µg/plate). All the t/c values were much lower than those obtained using a positive control (sodium azide, 1 µg/plate).

molecules

MDPI

(µg/plate)	HU (mM)	C1 (mM)	C2 (mM)	C3 (mM)	C4 (mM)	C5 (mM)	C6 (mM)	C7 (mM)	C8 (mM)
**1**	0.0333	0.0224	0.0228	0.0257	0.0290	0.0233	0.0226	0.0217	0.0206
**5**	0.1665	0.1120	0.1138	0.1284	0.1448	0.1164	0.1130	0.1083	0.1029
**10**	0.3330	0.2240	0.2276	0.2568	0.2895	0.2328	0.2260	0.2165	0.2058
**50**	1.6650	1.1198	1.1378	1.2839	1.4476	1.1642	1.1301	1.0826	1.0292
**100**	3.3300	2.2396	2.2755	2.5677	2.8953	2.3283	2.2603	2.1652	2.0585

Table S10. Concentrations tested for all the samples (1, 5, 10, 50, 100 µg/plate) related to the respective molar concentrations.
